# The Study and Practice of French in School

**Published:** 1897-08

**Authors:** 


THE STUDY AND PRACTICE OF FRENCH IN SCHOOL. Natural method on a new plan, with thorough drill in pronunciation, by Louise C. Boname, teacher of the French language and literature. Philadelphia, 258 South Sixteenth Street,

1896. In three parts. Part I, price, cloth, 60 cents; part II, price, cloth, 90 cents; and part III, price, cloth, $1.

Mlle. Boname is well known and highly regarded in Philadelphia among educators and parents as a most successful and talented teacher of her specialty—the French language. Her management of pupils and personal manners toward them in the school-room are highly commended. It is not surprising, therefore, that with her ripe experience that she should devise a method of her own for inducting youth into the knowledge of the mysteries of the French tongue.

This method has now been crystallized, so to speak, in type, and the product is before us in the three small volumes to which we now direct attention.

Part I is for beginners. Familiar subjects have been selected, their French names given, and the pupil proceeds at once to exercises which put into use the words he or she has just learned. The stories of Red Riding Hood and Cinderella are incorporated, a few sentences at a time, into each lesson, and the individual words are treated and the principles governing their pronunciation elucidated. This first part is, indeed, given over mostly to the teaching of the principles of pronunciation. They are very carefully graduated, and are, therefore, no tax upon the minds of young pupils.

Part II, for intermediate classes. This volume teaches elementary grammar, and is, of course, an expansion of Part I. Conjugation of verbs is one of the most important and valuable parts of the book. It runs all through it. While teaching other parts of speech the pupil's attention is constantly recalled to some form of conjugation or some tense of the regular verb that it may the more deeply be impressed upon the memory. Only regular verbs are taught in this part.

Part III, for advanced classes. This teaches all the intricacies of the irregular verbs, all the peculiar idioms, and the syntax of the French language. Here, too, is careful gradation of exercises, thorough analysis, continued repetition. This member of the series is made highly interesting to the pupil, and hence must the lessons taught the more readily enter the mind and remain there.

Any person, young or old, who will faithfully follow and thoroughly learn the lessons contained in these three parts will be certain to acquire an enviable knowledge of French. Indeed, we do not see how they can help learning with such instruction as is contained in these three admirable little books.

